# Neural Evidence of Hierarchical Cognitive Control during Haptic Processing: An fMRI Study

**DOI:** 10.1523/ENEURO.0295-18.2018

**Published:** 2018-11-27

**Authors:** Ane Gurtubay-Antolin, Patricia León-Cabrera, Antoni Rodríguez-Fornells

**Affiliations:** 1 Cognition and Brain Plasticity Group, Bellvitge Biomedical Research Institute (IDIBELL), Barcelona 08097, Spain; 2 Department of Cognition, Development and Education Psychology, Campus Bellvitge, University of Barcelona, Barcelona 08907, Spain; 3 Institute of Research in Psychology (IPSY) and in Neuroscience (IoNS), Université catholique de Louvain, 1348, Louvain la Neuve, Belgium; 4 Catalan Institution for Research and Advanced Studies (ICREA), Barcelona 08010, Spain

**Keywords:** cognitive control, haptic, manipulation, SI, somatosensory predictions, supramarginal gyrus

## Abstract

Interacting with our immediate surroundings requires constant manipulation of objects. Dexterous manipulation depends on comparison between actual and predicted sensory input, with these predictions calculated by means of lower- and higher-order corollary discharge signals. However, there is still scarce knowledge about the hierarchy in the neural architecture supporting haptic monitoring during manipulation. The present study aimed to assess this issue focusing on the cross talk between lower-order sensory and higher-order associative regions. We used functional magnetic resonance imaging in humans during a haptic discrimination task in which participants had to judge whether a touched shape or texture corresponded to an expected stimulus whose name was previously presented. Specialized haptic regions identified with an independent localizer task did not differ between expected and unexpected conditions, suggesting their lack of involvement in tactile monitoring. When presented stimuli did not match previous expectations, the left supramarginal gyrus (SMG), middle temporal, and medial prefrontal cortices were activated regardless of the nature of the haptic mismatch (shape/texture). The left primary somatosensory area (SI) responded differently to unexpected shapes and textures in line with a specialized detection of haptic mismatch. Importantly, connectivity analyses revealed that the left SMG and SI were more functionally coupled during unexpected trials, emphasizing their interaction. The results point for the first time to a hierarchical organization in the neural substrates underlying haptic monitoring during manipulation with the SMG as a higher-order hub comparing actual and predicted somatosensory input, and SI as a lower-order site involved in the detection of more specialized haptic mismatch.

## Significance Statement

The findings in the present study have important implications for the understanding of the neural architecture that supports haptic monitoring during manipulation. The results point, for the first time, to a hierarchical organization in the neural substrates underlying haptic monitoring during manipulation. In this hierarchy, the supramarginal gyrus (SMG) is positioned as a higher-order region comparing predicted and actual somatosensory input, and primary somatosensory area (SI) as a lower-order site involved in the detection of more specialized haptic mismatches. The increased functional connectivity between the SMG and SI during the processing of unexpected stimuli emphasizes the cross talk between lower-order sensory and higher-order associative regions during manipulation.

## Introduction

Interacting with our immediate surroundings requires constant manipulation of objects. Dexterous manipulation (sequences of somatosensory events linked to subgoals) requires that monitoring mechanisms (controllers) adapt motor commands to the relevant physical properties of the target object (Johansson and Westling, 1988). Motor commands are based on both the previously stored sensorimotor memory representations of objects (Johansson and Cole, 1992) and the current state of the motor apparatus (which depends on previous representations of the body in terms of proprioceptive information; Giummarra et al., 2008). Furthermore, information about the surface properties of the object is provided by somatosensory afferents in the hand. In particular, this relies mainly on the timing and firing rate of slow- and fast-adapting afferent populations (FA-I and SA-I) that can discriminate different surface curvatures after as few as five afferents have begun firing (Johansson and Westling, 1987; Johansson and Birznieks, 2004).

To regulate haptic monitoring, controllers compare the actual sensory input with the expected sensory consequences of initiated motor commands by means of the efference copy (Feinberg, 1978; Wolpert and Miall, 1996; Frith, 2014). This copy of efferent motor commands allows calculating how self-generated movement might influence the input sensory signal as well as maintaining performance in the presence of feedback delays. In other words, these predictions are necessary, taking into account that sensorimotor monitoring loops involved in corrective actions have long time delays (∼100 ms; Johansson and Flanagan, 2008). Of note, in addition to the efference copy (where a copy of the motor commands issued to an effector is projected to low-level somatosensory neurons; Von Holst and Mittelstaedt, 1950), the information transfer from motor to sensory areas might also occur at multiple levels, leading to the distinction of lower- and higher-order (corollary discharge) CD signals (Crapse and Sommer, 2008). Lower-order CD signals regulate the sensory information that enters the system whereas higher-order CD signals implement adjustments in anticipation of the sensory input, facilitating the contextual interpretation of sensory information. to combine these two types of signals, the interplay between lower-order sensory areas and higher-order associative regions is crucial. Despite previous literature supporting the hierarchical nature of haptic processing (Bodegård et al., 2001; Bohlhalter et al., 2002; Sathian et al., 2011; Kassuba et al., 2013), there is still scarce knowledge regarding the hierarchy of the neural substrates underlying haptic monitoring and how these two types of information are implemented by local and global networks.

The present study assessed this issue by focusing on the cross talk between lower-order somatosensory and higher-order associative regions. To address this, we first identified, using an independent localizer task, lower-order somatosensory regions that process shapes or textures selectively. Afterward, we conducted a haptic discrimination task in which unexpected shapes and textures were presented to trigger monitoring mechanisms involved in the comparison between predicted and actual sensory input. We assessed whether the specialized haptic areas identified with the localizer task distinguished expected from unexpected (50%) stimuli in the category they were suited to process. We also conducted a whole-brain analysis to identify higher-order areas involved in haptic monitoring. We hypothesized that higher-order associative regions supporting haptic monitoring during manipulation would respond similarly regardless of the nature of the haptic mismatch, independently of whether it was a shape or texture mismatch. Some of these areas (the highest in the hierarchy) might even serve to detect mismatches in other sensory modalities. In contrast, we expected to see different patterns of response in specialized somatosensory regions (e.g., somatosensory cortices or areas selectively responding to haptic exploration of shapes and textures) depending on the type of tactile property (shape or texture) violating the expectation.

## Materials and Methods

### Participants

Twenty-two right-handed participants (13 female, mean age = 23.2 ± 1.4 years) took part in the experiment. For the localizer task, the data of 20 subjects were analyzed, since two subjects were excluded from the fMRI analysis. One subject was excluded due to anomalous cortical response to tactile events, which did not elicit activity in the somatosensory cortex. Moreover, the logfile of another participant was not generated. For the haptic discrimination task, three additional subjects were excluded from the fMRI analysis since their responses were not recorded. Hence, the fMRI analysis of the haptic discrimination task included seventeen participants (12 female, mean age = 23.4 ± 1.5 years). The experiment was undertaken with the understanding and written consent of each participant and was approved by the local ethics committee in accordance with the Declaration of Helsinki.

### Procedure and general experimental design

Before the single scanning session participants underwent a training phase that lasted ∼30 min. During the training phase, they became familiarized with the haptic stimuli (used in both the independent localizer task and the haptic discrimination task) and the experimental procedure. Since inside the scanner all the instructions were vocally presented, the meaning of each auditory cue was explained, and a brief simulation was conducted. Participants lay supine in the scanner and were blindfolded during the entire scanning session. First, the T1 structural image was acquired. To avoid circularity (Kriegeskorte et al., 2008), an independent functional localizer task was then conducted to localize areas typically involved in haptic processing shape and texture [from here on referred to as property-selective haptic regions of interest (ROIs); Amedi et al., 2001]. Lastly, the haptic discrimination task was conducted, to assess whether the previously identified haptic-specialized areas distinguished expected from unexpected stimuli and to establish higher-order regions involved in the detection of haptic incongruencies. Note that these higher-order areas are not selective to haptic processing and thus could not be spotted by the independent localizer task.

#### Independent functional localizer task

##### Stimuli

**Figure 1.**
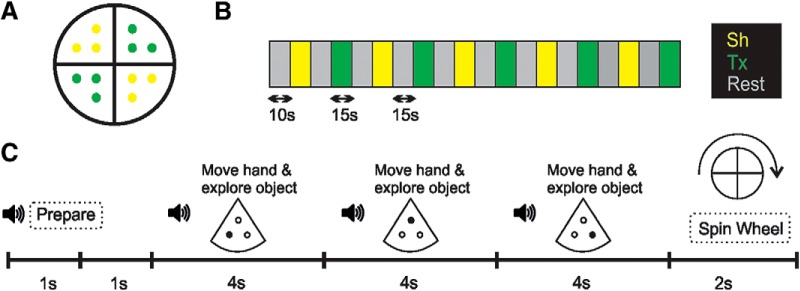
Stimuli and procedure used in the localizer task. ***A***, Stimuli: rotating tray with three objects (shapes: yellow) or three textures (green) in each quarter. Shapes were real 3D objects: pen cap, thread spool, eye drop bottle, clothespin, bottle cork, and mini pencil. Textures, ∼4 × 4 cm, were corduroy, cork, sackcloth, sandpaper, sponge, and scourer. ***B***, Block design for the single run. The run comprised 10 haptic blocks (yellow-shape; green-texture) and began with a 10-s interval that was not analyzed. The order of the haptic blocks was alternated. ***C***, Procedure for a haptic block. The block began with an auditory cue indicating that the haptic block would begin. After 1 s of silence, the first auditory cue indicated to the participant the first (of the sequence of three) stimulus and to palpate it. After 4 s of exploration, a second (and third) auditory cue indicated to move the hand toward and palpate the second (and third) stimulus. The hand always moved in the same direction when ranging from one stimulus toward the next one since the location of the stimuli was previously known. To conclude, an auditory cue instructed the participants to spin the tray (to prepare it for the next block) and rest.

For the localizer task, we used six real 3D objects (pen cap, thread spool, eye drop bottle, clothespin, bottle cork, and mini pencil) and six textures, ∼4 × 4 cm (corduroy, cork, sackcloth, sandpaper, sponge, and scourer), presented in a rotating tray (Fig. 1*A*).

##### Design

Participants were asked to palpate sequences of shapes or textures presented in a rotating tray and covertly recognize them. The task began with a 10-s resting interval that was not analyzed. We used a block design that consisted of a single run with two haptic conditions (shape, Sh; texture, Tx) and a rest condition (Fig. 1*B*). All blocks lasted 15 s, and Sh and Tx blocks were alternated between each pair of Rest blocks. Each haptic block consisted in palpating (4 s/palpation) three stimuli placed in a quarter of the rotating tray and then spinning the tray to continue with the next quarter (after the resting period; Fig. 1*C*). The hand always palpated the sequence of objects in the same order since the location of the stimuli was previously known. The task lasted ∼5 min.

#### Haptic discrimination task

##### Stimuli

A total of 25 3D haptic stimuli were manufactured, measuring ∼4 × 4 × 2 cm and varying in shape and texture. The 25 stimuli comprised a variety of five shapes (flower, circle, heart, square, and star) that were covered with five textures (corduroy, sandpaper, plastic, paper, and expanded polystyrene; Fig. 1*A*). Thus, each stimulus had two properties: a particular shape and a particular texture (e.g., circle of corduroy, circle of paper, etc.). All the stimuli were chosen so as to be easily identifiable, and the five shapes were previously used in two other studies (Gurtubay-Antolin et al., 2015; Gurtubay-Antolin et al., unpublished observation).

##### Design

Participants were presented with brief sequences of objects or textures to palpate and had to judge whether a touched stimulus corresponded to an expected stimulus whose name had been previously presented orally. Subjects were placed with the right hand facing upward, and the stimuli were placed into the subject’s palm by the experimenter. The experiment was designed as a mixed block/event-related design with two haptic conditions (shape, Sh; texture, Tx) in which subjects were instructed to attend to the shape or the texture of the stimuli (block design), a control motor condition (motor) in which they were asked to move their fingers as they were exploring an imaginary object (and were explicitly instructed not to touch themselves), and a “rest” condition.

**Figure 2.**
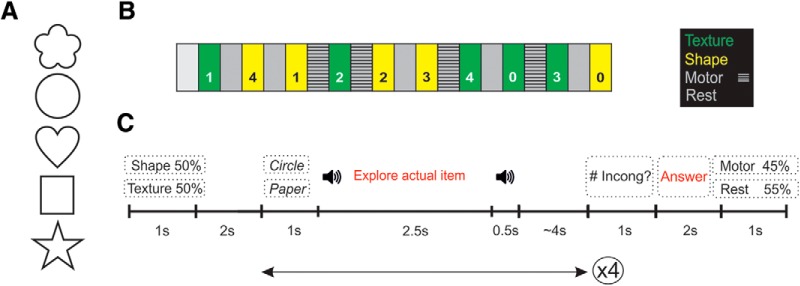
Stimuli and procedure used in the haptic discrimination task. ***A***, Stimuli: variety of five shapes (flower, circle, heart, square, and star). The 25 haptic stimuli were manufactured covering each of the five shapes with five textures (corduroy, sandpaper, plastic, paper, and expanded polystyrene). ***B***, Block design for one run. Each run comprised 10 haptic blocks (green-attend texture; yellow-attend shape) and began with a 10-s interval that was not analyzed. The order of the haptic blocks was randomized. Between two haptic blocks, rest or motor blocks were presented in randomized order. In each haptic block, four haptic trials where presented (see panel ***C*** for further information on haptic blocks). The number in each block corresponds to the number of incongruent trials presented in that block (ranging from a minimum of 0 to a maximum of 4). For a particular dimension (shape/texture), the number of incongruent trials within each block was not repeated in that run. ***C***, Procedure for a haptic block. The block began with an auditory cue instructing as to the dimension of the object that had to be attended to (50% shape, 50% texture). After a 2-s interval, the first haptic trial started. In each haptic trial, the name of an expected item was presented and an auditory cue indicated that the exploration of the actual item could begin. In half of the trials, the word delivered by the headphones corresponded to the touched object (congruent), and in the other 50% of trials, the object did not match the name (incongruent). We therefore had six conditions [four corresponding to all the possible combinations: congruent shape (CSh), incongruent shape (ISh), congruent texture (CTx), and incongruent texture (ITx); and two additional control conditions: motor (M) and rest (R)]. Free exploration was allowed. 2.5 s later, a second auditory cue indicated that the exploration period was over. After a jittering period, the next haptic trial could start. After four haptic trials, the participants were asked about the number of incongruent trials. Using their left hand, participants had to push a button as many times as the incongruent trials that had been presented (ranging from 0 to 4). To conclude, an auditory cue indicated whether a rest or “motor” block followed (a block in which participants were asked to move the fingers as they were exploring an imaginary object).

The experimental design consisted of four runs and each run comprised 10 haptic blocks (five Sh, five Tx), four motor blocks, and five rest blocks (Fig. 2*B*). All the runs began with a 10-s resting interval that was not analyzed. The type of haptic block (shape or texture) was randomized and lasted 32 s. Each haptic block consisted of four consecutive somatosensory trials (8 s each if we take into account the ∼4-s jittering interval). Each haptic block began with an auditory cue indicating the dimension of the object that had to be attended (50% shape, 50% texture). After a 2-s interval, the first haptic trial started (Fig. 2*C*). In each haptic trial, the name of an expected stimulus was vocally presented and a second later, an auditory cue indicated that the exploration of the actual stimulus could begin. In half of the trials, the word delivered by the headphones corresponded to the touched object (congruent, C), and in the other 50% of trials the object did not match the name (incongruent, I). All the possible combinations were presented the same number of times. We therefore had six conditions [four corresponding to all the possible combination: congruent shape (CSh), incongruent shape (ISh), congruent texture (CTx), and incongruent texture (ITx); and two additional control conditions: motor (M) and rest (R)]. Free exploration was allowed. 2.5 s later, a second auditory cue indicated that the exploration period was over. After a jittering period ranging from 2 to 6 s in 100-ms intervals (mean = 4 s), the next haptic trial could start. After four haptic trials, the subjects were asked about the number of incongruencies detected in the preceding block. Using their left hand, participants had to push a button as many times as there were identified incongruent trials (ranging from 0 to 4). For a particular dimension (Sh or Tx), the number of incongruent trials within each block was not repeated in that run. Between two haptic blocks, 10 s rest or motor blocks were presented in randomized order.

### Behavioral analysis

We considered a correct response block each time the number of incongruencies presented in a haptic block and the number of times the participant had pushed the button matched. The percentage of correct responses served (1) to rule out blocks with erroneous responses from the fMRI analysis, and (2) to obtain an overall estimate of the performance.

### Image acquisition

Scanning was performed on a 3-T Siemens Trio System. Functional data were acquired using a gradient echo pulse sequence (32 transverse slices oriented along the anterior-posterior commissural axis with a 30° upward tilt to avoid the eyes, repetition time of 2 s, echo time of 30 ms, 3 × 3 × 3.5 mm voxels, 0.8-mm interslice gap). A high-resolution T1-weighted magnetization-prepared rapid acquisition gradient echo (MPRAGE) image (240 slices sagittal, TR = 2300 ms, TE = 2.98 ms, 1-mm isotropic voxels) was also collected.

### Image processing and statistical analysis

fMRI data were analyzed using standard procedures implemented in the Statistical Parametric Mapping software (SPM8, Wellcome Trust Center for Neuroimaging, University College, London, United Kingdom; https://www.fil.ion.ucl.ac.uk/spm/). The preprocessing consisted of several steps: realignment, segmentation, normalization, and smoothing. To correct head-movement artefacts based on an affine rigid body transformation, images were spatially realigned with respect to the first volume of the first run (Friston et al., 1996). Each participant’s MPRAGE scan was coregistered to the mean echo-planar imaging (EPI) volume, produced in the previous step during spatial realignment. Each coregistered structural scan was then segmented using New Segment (Ashburner and Friston, 2005). Then the flow fields containing the deformation parameters of this template were used to normalize each participant’s realigned EPIs to MNI space. Finally, normalized EPI images were re-sliced to 2 × 2 × 2 mm and smoothed with an 8 mm FWHM Gaussian kernel (Ripollés et al., 2014, 2016).

#### Independent functional localizer task

We aimed to identify specialized haptic regions responding differentially to the exploration of shapes and textures (property-selective haptic ROIs) to analyze their involvement in tactile monitoring. To avoid circularity (Kriegeskorte et al., 2008), the ROIs were created using independent data obtained from the independent localizer task. For the independent functional localizer task, a block design matrix was specified using the canonical hemodynamic response function. Block onsets were modeled at the moment at which participants heard the auditory cue that indicated that they could start palpating the first of the three stimuli placed in a quarter of the rotating tray. First-level statistical analysis was based on a least square estimation using the general linear model. Individual brain responses to the “shape” and “texture” conditions were modeled with a regressor wave form convolved with a canonical hemodynamic response function. Movement parameters (estimated during the realignment phase) were also included in the model as covariates of no interest to correct for motion effects as well as as constant vectors. Linear contrast images for the main effect of haptic processing (Sh + Tx > rest) and the main effect of property (Sh > Tx and vice versa) were calculated for each subject, and statistical parametric maps (SPMs) were generated. Group activation was calculated using a random effects model, accounting for intersubject variance. Main effects of haptic processing were only used for sanity checks to confirm that they activated sensorimotor cortical areas. Property-selective haptic ROIs were created entering individual contrast images into a second level one-sample *t* test to test for (1) main effects of shape (Sh > Tx) and (2) main effects of texture (Tx > Sh; *p*_uncorrected_ = 0.005, k = 50). To maximize sensitivity within our ROIs, we performed a meta-analysis using NeuroSynth [a platform for large-scale, automated meta-analysis of fMRI data; http://www.neurosynth.org; Yarkoni et al., 2011; for a similar approach, see Ripollés et al., 2016]. We put together a term-based search for “tactile” that resulted in 190 studies (search performed on October 20, 2016). Then, a forward inference mask (which represented the probability that the term tactile was associated with a particular activation) was generated (corrected at *p*_FDR_ = 0.01). We then refined the previously created ROIs by masking them with the results of the NeuroSynth meta-analysis. In other words, each final ROI contained only voxels that were part of the original ROIs and that were also tactile-related according to the meta-analysis.

#### Haptic discrimination task

For the haptic discrimination task, an event-related design matrix was specified using the canonical hemodynamic response function. Trial onsets were modeled at the moment at which participants heard the auditory cue that indicated that they could palpate the stimuli. We only analyzed blocks with correct responses (blocks where the number of presented incongruencies matched the times the participant pushed the response button). Blocks with incorrect responses were not analyzed. First-level statistical analysis was based on a least square estimation using the general linear model. Individual brain responses to the different conditions [congruent shape (CSh), incongruent shape (ISh), congruent texture (CTx), incongruent texture (ITx), and motor (M)] were modeled with a regressor wave form convolved with a canonical hemodynamic response function. Movement parameters (estimated during the realignment phase) were also included in the model as covariates of no interest to correct for motion effects as well as constant vectors.

### ROI analysis

First, a ROI analysis was performed using the results from the independent functional localizer task. To assess whether property-selective haptic ROIs could detect conflicting haptic information in the category they were suited to process, paired t *tests* were conducted. We compared mean beta values (1) between CSh and ISh conditions in ROIs selective to shape processing and (2) between CTx and ITx in ROIs selective to texture processing. The significance threshold was corrected for multiple comparisons taking into account the number of ROIs.

### Whole-brain analysis

Additionally, to identify higher-order areas involved in haptic monitoring, a whole-brain analysis was conducted. Linear contrast images for (1) the main effect of haptic processing with the related motor component (CSh + ISh + CTx + ITx > R), (2) motor processing without somatosensory stimulation (M > R), (3) somatosensory stimulation without motor processing (CSh + ISh + CTx + ITx > M), (4) the main effect of shape (CSh + ISh > CTx + ITx), and (5) the main effect of texture (CTx + ITx > CSh + ISh) were calculated to perform some sanity tests. These tests aimed to confirm that the previous contrasts activated (1) sensorimotor cortical areas, (2) motor, but not somatosensory, areas, (3) somatosensory, but not motor, areas, and (4) previously reported areas specifically processing shapes as opposed to textures and (5) vice versa.

To identify regions involved in haptic monitoring, linear contrast images for (1) the main effect of congruency (CSh + CTx > ISh + ITx), (2) the main effect of incongruency (ISh + ITx > CSh + CTx), and the interaction terms (3) for greater shape incongruency (ISh – CSh > ITx – CTx), and (4) for greater texture incongruency (ITx – CTx > ISh – CSh), were calculated for each subject. SPMs were generated. Group activation was calculated using a random effects model, accounting for intersubject variance. To test for the main effect of incongruency (ISh + ITx > CSh + Ctx and the reverse) and the interaction term (ISh – CSh > ITx – CTx and the reverse), the individual contrast images were entered into a second level repeated measures ANOVA with two within-subjects factors (property and congruency) and two levels each [shape, texture (Sh and Tx) and congruent, incongruent (C and I), respectively]. The results were thresholded at *p* = 0.05 FEW-corrected at cluster level, with a cluster-forming (voxel-wise) threshold of *p*_uncorrected_ < 0.001 (Woo et al., 2014; Flandin and Friston, 2017). In cases where clusters were further FWE-corrected at the voxel-level, the voxel-level and the FWE-corrected *p* value are explicitly mentioned.

### Functional connectivity (psychophysiological interaction; PPI)

PPI analyses (Friston et al., 1997; Gitelman et al., 2003) identify voxels in which activity is more closely related to activity in a seed ROI in a given psychological context. In the present study, PPI analyses were performed to identify brain regions that were more functionally coupled (1) with regions that showed the main effects of incongruency (from now on referred to as iROIs) while processing incongruent versus congruent trials, (2) main effects of congruency (cROIs) while processing congruent versus incongruent trials, and (3) an interaction between incongruency and property (IntROIs) while processing shape incongruencies in the opposite direction of texture incongruencies (ISh – CSh > Itx – CTx).

Four mm radius spheres were created around the group peaks obtained for (1) iROIs, (2) cROIs, and (3) IntROIs. For all participants, individual deconvolved time-series were extracted from all voxels within the seed ROIs. New linear models were generated at the individual level, using three regressors. The first regressor was the activity extracted in the seed area. The second regressor represented the condition as a vector that coded (1) the main effect of incongruency (CSh: –1, ISh: 1, CTx: –1, ITx: 1) for iROIs, (2) the main effect of congruency (CSh: 1, ISh: –1, CTx: 1, ITx: –1) for cROIs, and (3) the incongruency × property interaction (CSh: –1, ISh: 1, CTx: 1, ITx: –1) for IntROIs. The third regressor represented the interaction of interest between the ﬁrst (physiologic) and the second (psychological) regressors. This was calculated as the element by element product of the extracted time-series (the first eigenvariate from each voxel in the sphere) and the second regressor. The result of this product was then reconvolved with the canonical hemodynamic response function to create the final PPI regressor (Gitelman et al., 2003). The design matrix also included movement parameters as a regressor of no interest. A signiﬁcant PPI indicated a change in the regression coefﬁcients between any reported brain area and the seed area, related to the experimental condition (ISh + ITx > CSh + Ctx for iROIs; CSh + CTx > ISh + Itx for cROIs; and ISh – CSh > Itx – CTx for IntROIs). The voxels identiﬁed in this analysis show a pattern of activity correlated with the seed region. Individual summary statistic images obtained at the ﬁrst level (ﬁxed effects) analysis were entered in a second-level (random effects) analysis using a one-sample *t* test. The results were thresholded at *p* = 0.05 FEW-corrected at cluster level, with a cluster-forming (voxel-wise) threshold of *p*_uncorrected_ < 0.001 (Woo et al., 2014; Flandin and Friston, 2017).

## Results

### Behavioral results

Overall percentage of correct response was 91% (SD = 5; considering both types of haptic blocks). The accuracies considering each property separately were 92 ± 5% for shape blocks and 90 ± 9% for texture blocks, with no significant difference between the two (*t*_(16)_ = 0.1; *p* = 0.3).

### fMRI results

#### Property-selective haptic ROI creation from localizer task

**Figure 3.**
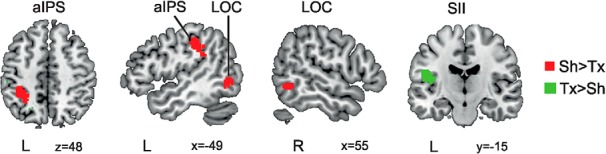
Property-selective haptic ROI creation from localizer task. In red, regions more active during the haptic shape than texture processing (left anterior intraparietal sulcus -aIPS- and bilateral lateral occipital complex -LOC-). In green, regions more active during the haptic texture than shape processing (left secondary somatosensory area -SII- , parietal operculum).

The results revealed a main effect of shape (Sh vs Tx) in three clusters located in the left anterior intraparietal sulcus [aIPS; –34 –40 46, *t*_(19)_ = 5.49, k = 53044], in the left lateral occipital complex [LOC; –48 –66 –6, *t*_(19)_ = 3.65, k = 6044], and in the right LOC [52 –56 –6, *t*_(19)_ = 3.10, k = 3324]. Additionally, a cluster located in the left secondary somatosensory area (SII), parietal operculum [–42 –18 24, *t*_(19)_ = 5.01, k = 197] showed a main effect of texture (Tx vs Sh; Fig. 3).

#### Haptic discrimination task

The average number of trials included in the fMRI analysis was 36 ± 1 (mean ± SD, CSh 37 ± 2; ISh 36 ± 3; CTx 35 ± 5; ITx 35 ± 3). The haptic discrimination task activated a network associated with haptic stimulation, comprising bilateral sensorimotor areas, insulae, posterior parietal cortices, LOCs, premotor and supplementary motor areas, prefrontal regions, and thalamus.

### Incongruence in property-selective haptic ROIs

Results of paired t *tests* between the CSh and ISh conditions for the three ROIs showing a main effect of shape (Sh vs Tx) were not significant [left aIPS (*p* = 0.2), left LOC (*p* = 0.2), right LOC (*p* = 0.3), with the significance threshold *p*_BONFERRONI_ = 0.05/3 = 0.017]. Results of a paired *t* test between the CTx and ITx conditions for the ROI showing a main effect of texture [Tx vs Sh; left SII, parietal operculum (*p* = 0.06)] were not significant, either.

### Whole-brain analysis: flexible factorial results

**Figure 4.**
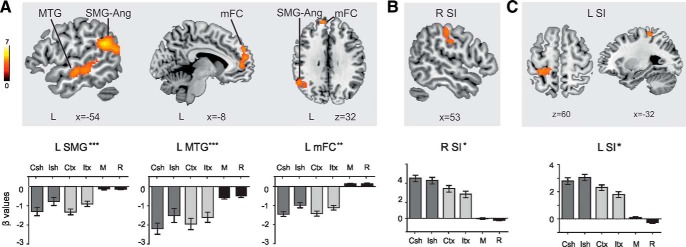
***A***, Regions active during haptic incongruency revealed by contrasting incongruent (ISh+ITx) versus congruent (CSh+CTx) trials. ***B***, Region active during haptic congruency revealed by contrasting congruent (CSh+CTx) versus incongruent (ISh+ITx) trials. ***C***, Region showing a different pattern of response to shape and texture incongruencies revealed by contrasting shape incongruency (ISh–CSh) versus texture incongruency (ITx–CTx) trials. Bar graphs on the lower row show mean beta coefficients within ROIs for each condition of interest (CSh congruent shape, ISh incongruent shape, CTx congruent texture, ITx incongruent texture; M motor, movement without haptic input; R, rest). Ang, angular gyrus; mFC, medial frontal cortex; MTG, middle temporal gyrus; SI, primary somatosensory cortex; SMG, supramarginal gyrus; R, right hemisphere and L, left hemisphere. MNI coordinates. Maps are thresholded at ***p FWE cluster-level = 0.001, **p = 0.005, *p = 0.05 (with cluster-forming voxel-wise thresholds of *p*_uncorrected_ < 0.001) and a minimum cluster size of 50 voxels. Note that the clusters in the SMG and MTG are further FWE-corrected at *p* < 0.05 at the voxel-level.

Flexible factorial results revealed important effects of incongruency in a cluster located in the left supramarginal (SMG; and part of angular) gyrus [iROI1; –54 –52 24, *t*_(48)_ = 5.44, *p_FWE_*_cluster_ < 0.001, *p*_FWE voxel_ = 0.03, k = 984], in the left middle temporal gyrus [MTG; iROI2; –48 –18 –10, *t*_(48)_ = 5.31, *p*_FWE cluster_ < 0.001, *p*_FWE voxel_ = 0.04, k = 744], and in the medial prefrontal cortex [mPFC; iROI3; –10 54 34, *t*_(48)_ = 4.64, *p*_FWE cluster_ = 0.003, k = 421; Fig. 4*A*]. An important effect of congruency was found in the right primary somatosensory area [SI; cROI; 58 –16 32, *t*_(48)_ = 4.42, *p*_FWE cluster_ = 0.04, k = 235; Fig. 4*B*] as well as a significant interaction of incongruency × property (ISh + ITx > CSh + Ctx) in the left SI [IntROI; –32 –36 64, *t*_(48)_ = 4.39, *p*_FWE cluster_ = 0.03, k = 238; Fig. 4*C*].

### Functional connectivity results

**Figure 5.**
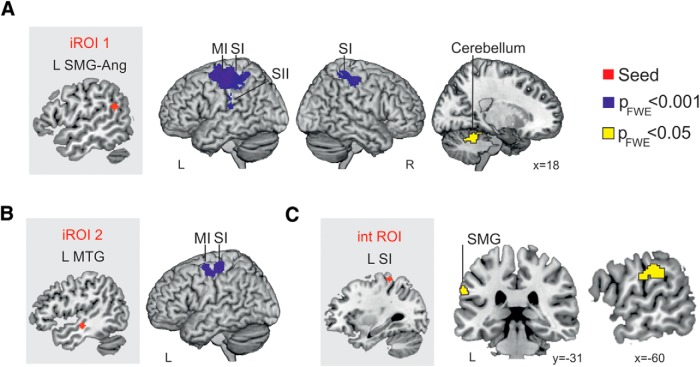
Functional connectivity results (PPI). ***A***, Connectivity results seeding from ROI that showed main effects of incongruency (iROIs) while processing incongruent versus congruent trials (CSh: –1, ISh: 1, CTx: –1, and ITx: 1). Seeds are 4**-**mm spheres centered at the peak coordinate of the cluster located in left supramarginal-angular gyrus (iROI1) and in left middle temporal gyrus (iROI2; ***B***). ***C***, Connectivity results seeding from the ROI showing an interaction between incongruency and property (IntROI), while processing shape incongruencies different from texture incongruencies (CSh: –1, ISh: 1, CTx: 1, and ITx: –1). Maps are thresholded at *p* FWE cluster-level = 0.001 and *p* = 0.05 (with cluster-forming voxel-wise thresholds of *p*_uncorrected_ < 0.001) and a minimum cluster size of 50 voxels. MI, primary motor cortex; MTG, middle temporal gyrus; SI, primary somatosensory cortex; SII, secondary somatosensory cortex; SMG, supramarginal gyrus; R, right hemisphere and L, left hemisphere. MNI coordinates.

We ran PPI analyses to further investigate which brain regions showed a signiﬁcant change in the regression coefﬁcients between that area and the seed area, related to the experimental condition (ISh + ITx > CSh + Ctx for iROIs and ISh – CSh > Itx – CTx for Int ROI), indicating that they were more functionally coupled. The left SMG (iROI1) showed increased incongruency-related connectivity with a cluster that included the left primary motor area (MI), premotor area, SI, SII, and part of the superior parietal lobe [SPL; –32 –16 42, *t*_(16)_ = 6.21, *p*_FWE cluster_ < 0.001, k = 2045], as well as with a cluster including regions of the right SI and SPL [50 –42 66, *t*_(16)_ = 4.93, *p*_FWE cluster_ < 0.001, k = 451]. With a more liberal threshold of *p*_FWE cluster_ = 0.05, the left SMG also presented increased connectivity during incongruent trials with a region in the right cerebellum [–20 –48 –28, *t*_(16)_ = 5.04, *p*_FWE cluster_ = 0.01, k = 201; Fig. 5*A*]. Increased incongruency-related connectivity was also found between the left MTG (iROI2) and the left MI and SI [–46 –22 62, *t*_(16)_ = 5.22, *p*_FWE cluster_ < 0.001, k = 546; Fig. 5*B*]. The cluster at the left medial frontal cortex (iROI3) did not show any increased incongruency-related connectivity. The cluster in the right primary somatosensory area (cROI) did not show any increased congruency-related connectivity. Lastly, the left SI (IntROI) presented increased connectivity for higher shape incongruency than texture incongruency with the left SMG area [–62 –34 30, *t*_(16)_ = 5.98, *p*_FWE cluster_ = 0.05, k = 152; Fig. 5*C*].

For further information, unthresholded t-maps resulting from the fMRI analysis have been uploaded to NeuroVault (Gorgolewski et al., 2015; https://neurovault.org/collections/4161/).

## Discussion

The present study aimed to elucidate the hierarchy in the neural substrates underlying haptic monitoring during manipulation, focusing on the cross talk between the lower-order sensory regions and the higher-level associative areas implicated. We found that the aIPS, LOC, and SII responded differently to the exploration of shapes and textures, whereas they did not differ between expected and unexpected conditions. This suggests that they are specialized in haptic exploration and processing but are not involved in tactile monitoring. The left SMG, the middle temporal, and the medial prefrontal cortices were activated whenever there was a haptic mismatch, regardless of its nature (shapes and textures alike). In contrast, the activity in the left SI distinguished between unexpected shapes and textures, in line with more specialized haptic mismatch detection. Moreover, the left SMG-Ang area and left SI were more functionally coupled during unexpected trials.

The activity observed in the left SMG-Ang gyrus for unexpected haptic input converges with lesion and functional neuroimaging studies relating this area to comparison processes between predicted and actual sensory consequences of ongoing actions (Sirigu et al., 2004; Desmurget et al., 2009). Prior electrophysiological studies found that the mismatch between internal predictions and reafferent signals elicited a parietally distributed error signal resembling the N400 component (Gurtubay-Antolin et al., 2015; Padrao et al., 2016), whose main neural substrate has been located in the SMG (Lau et al., 2008; Baumgaertner et al., 2002). The functional connectivity analyses revealed that the left SMG-Ang was more functionally coupled during unexpected than expected trials with bilateral sensorimotor regions and the right cerebellum, suggesting a pivotal role of the SMG-Ang area in orchestrating the monitoring of sensory predictions. In this vein, Jenmalm et al. (2006) observed activation in the right SMG area when unexpectedly heavy and light weights were lifted, as well as activity in the left SI and right cerebellum during the lifting of unexpectedly heavy and light weights, respectively. Taken together, these results favor the idea that the controller (SMG-Ang) compares the actual sensory input projected by the early somatosensory cortex with the predictions computed in the cerebellum, made on the basis of proprioceptive information. In fact, the cerebellum is thought to be the keystone computing these predictions since it is widely accepted that it contains internal models of the motor apparatus (Wolpert et al., 1998) and is involved in recruiting internal representations of object properties (Bursztyn et al., 2006).

Furthermore, the preponderant left-lateralized (contralateral) activity of the SMG-Ang when manipulating objects with the right hand contrasts with the study by Jenmalm et al. (2006), who reported activity in the right SMG even when people used the right hand for lifting. Indeed, a general dominance of the right hemisphere for somatosensory functions has been proposed (Naito et al., 2005). However, aspects such as the selection of the hand configuration (Emmorey et al., 2007) might explain the left-lateralized preponderance, which converges with studies reporting greater activation of the left SMG when participants are asked to pantomime object use (Rumiati et al., 2004), or when deaf signers name objects compared to speakers (Emmorey et al., 2007). In this task, the representation of the expected object was available before its perception, so participants might have benefited from preparing a hand configuration specific for the expected stimulus.

Different patterns of response were found for unexpected shapes and textures in regions of the left SI, including areas that were functionally connected to the SMG area during incongruence. This supports the notion that a more specialized mismatch detection occurs in the left SI, in line with the view of SI as a lower-order region in the hierarchy of the neural substrates that underlie tactile monitoring. This finding emphasizes the importance of the contralateral SI not only in early somatosensory processing and short-term maintenance of haptic traces (Kaas et al., 2007; Romo et al., 1999), but also in haptic monitoring. In accordance with our results, SI has been seen to participate in decision-making during the haptic choice in a haptic delay task (Wang et al., 2012). SI cells showed differential neural activity when monkeys had to choose between different haptic objects, and such differential activity diminished significantly in erroneous trials. In addition, our results show increased functional connectivity between SI and a region in the SMG when unexpected shapes but not textures were presented, pointing to a relevant cross talk between these regions. However, the right SI did not show increased functional connectivity related to incongruence, highlighting the notion that the functional connections between the left SI and the SMG region are uniquely enhanced by incongruence processing. On the whole, this interplay seems to be important in the detection of haptic expectancy violations, with SI processing more specialized tactile information than the SMG-Ang area.

Lastly, the results suggest that the MTG and the mPFC are additional higher-order regions that work in parallel with the SMG-Ang. During mismatches, the MTG showed increased functional connectivity with the left sensorimotor cortex. The MTG has been observed to respond to deviant stimuli in a tactile oddball task (Allen et al., 2016) and to unexpected touch sensations in monkeys (Perrett et al., 1990); thus, it seems to be related to violations of predictions selective to the haptic domain. Moreover, the activation found in the mPFC seems to correspond to higher-order cognitive control areas (Rushworth et al., 2004; Nee et al., 2011). This area has been associated with mismatch detection in several sensory modalities, suggesting its multimodal or amodal nature (Gaebler et al., 2015; Blakemore et al., 2000; Malekshahi et al., 2016). This matches well with our results, which show a lack of functional connections between the mPFC and somatosensory areas (while these are present between the somatosensory regions and the SMG and MTG). There is still debate on this question, as some theories state that the mPFC supports conﬂict monitoring by calling for control processes to resolve discrepancies (Botvinick et al., 2001) while a recent line of research suggests that it responds to unexpectedness (Jessup et al., 2010; Oliveira et al., 2007).

Importantly, the fact that the SMG-Ang, the MTG and the mPFC exhibited negative values in all the haptic conditions might reflect the neuronal inhibition associated with the suppression of items that were not expected (Frankenstein et al., 2003). This inhibition was greater in congruent trials where the actual stimulus matched the expectancy, since the non-expected stimuli had to be inhibited for a longer period. In incongruent trials, the non-expected items were inhibited only until participants realized that the touched stimulus was not the expected one. Subsequently, they might have disinhibited the non-expected items to identify the actual item, even if this was not required in the task. An alternative explanation for these deactivations is that they are associated with the decrease in activity shown by areas of the default mode network (Xu et al., 2016). Recent findings suggest the existence of a gradient in the human cortical organization (which spans from primary sensorimotor cortices to higher order areas whose activity is not specific to a single sensory modality) that is reflected in cortical microstructure and macroscale connectivity (Huntenburg et al., 2018). According to this view, a continuous pattern of connectivity exists between sensorimotor areas that converge in multimodal integration areas, and higher order regions of the default mode network. Our results fit nicely with this interpretation.

Of note, the localizer task did not show shape-related activations in sites of the IPS that are typically involved (Sathian, 2016), nor texture-selective activity in the early visual cortex (Sathian et al., 2011; Eck et al., 2013). This raises questions about the actual sensitivity of the localizer task, which may have been affected by the limited amount of time for scanning. Despite this potential limitation, the task revealed property-selective haptic ROIs that fit nicely with previous reports of shape-selective activations in the LOC and the IPS (Roland et al., 1998; Sathian et al., 2011), as well as texture-sensitive areas located in the SII, parietal operculum (Roland et al., 1998). Particularly, the LOC and the IPS are involved not only in haptic perception but also in haptically-guided grasping. This is suggested by the fact that the occipital pole (active during haptic exploration of shapes) shows stronger functional connectivity with the LOC and the IPS during haptic than visual exploration (Monaco et al., 2017) and that the aIPS is sensitive to characteristics of the required grasp (Marangon et al., 2016).

Altogether, the results point to a hierarchical organization in the neural substrates underlying haptic monitoring during manipulation, with the SMG as a higher-order region comparing actual and predicted somatosensory input and SI as a lower-order site involved in the detection of more specialized haptic mismatch. We report, for the first time, the functional coupling of these regions during the processing of unexpected tactile stimuli, supporting their pivotal role in haptic monitoring.

## Synthesis

Reviewing Editor: Satu Palva, University of Helsinki Decisions are customarily a result of the Reviewing Editor and the peer reviewers coming together and discussing their recommendations until a consensus is reached. When revisions are invited, a fact-based synthesis statement explaining their decision and outlining what is needed to prepare a revision will be listed below. The following reviewer(s) agreed to reveal their identity: Daniel Margulies, Gregory Kroliczak.

In this fMRI study, neural underpinnings of haptic monitoring of shape or texture during (active tactile) stimulus manipulation, were studied. The main experiment involved processing of haptic mismatch studied in a discrimination task, but there was also an independent haptic localizer run and functional connectivity analyses to identify lower-level (sensory) and higher-level (associative) regions that demonstrated significant cross-talk during haptic manipulation. Whereas the independently identified “haptic areas” did not show sensitivity to stimulus mismatch (as if they were playing no role in haptic monitoring), the whole-brain analysis revealed three areas - the supramarginal gyrus (SMG), middle temporal, and medial prefrontal regions of the left hemisphere - sensitive to mismatch, regardless of its nature (i.e., whether related to shape or texture). Significant increase in connectivity was found between SMG and primary somatosensory cortex (SI). These results point to a hierarchical organization in the neural underpinnings of haptic monitoring during stimulus manipulation, with SMG being one of the most critical nodes. Both reviewers found your manuscript to be very clearly written, and establishing a straightforward justification for the experiments and analyses. There were some minor issues that were suggested to be corrected before publication.

Minor comments

- The location within the inferior parietal lobule is often referred to in the Discussion as SMG; however, it appears to also contain portions of the angular gyrus in Figure 5A (though unclear in Figure 5C). Would it be more appropriate to refer to the SMG as the SMG/Ang in the Discussion? If there is a motivation for this terminology, it would be helpful to mention that in the Discussion as well.

- The authors discuss the negative activation within the SMG-Ang as potentially reflecting neuronal inhibition. I would recommend the authors also consider the literature on the default mode network, which also incorporates this region. The numerous publications regarding task-induced deactivations, which include the regions mentioned here, may offer an additional perspective for interpreting the deactivations observed in the current study.

Along those lines, it is also noteworthy that all the regions presented in Figure 4a are included in the default-mode network. One recent interpretation of this networks functional role that the authors may feedback to sensorimotor systems (Huntenburg et al, TICS, 2018).

- Have the authors considered uploading their statistical maps to www.neurovault.org ? This would help researchers in the future to compare their findings to the maps produced in the current study, and would be particularly helpful for assessing converging findings.

- It would be informative to provide more details about “a jittering period (mean = 4 sec)”; were there 5 different intervals, like 3.5, 3.75, 4.0, 4.25, and 4.5 s or something else?

- Figure 3. Caption should have a header (title).

- Whereas your study is on the role of haptics in perception, there are some nice recent developments in the use of haptic domain for studying motor control, e.g., which would be worth of citing in the text.

(1) Monaco et al. (2017). Recruitment of Foveal Retinotopic Cortex During Haptic Exploration of Shapes and Actions in the Dark. J Neurosci, 37(48), 11572-11591. doi: 10.1523/JNEUROSCI.2428-16.2017

(2) Marangon et al. (2016). Haptically guided grasping. fMRI shows right-hemisphere parietal stimulus encoding, and bilateral dorso-ventral parietal gradients of object- and action-related processing during grasp execution. Front Hum Neurosci, 9, 691. doi: 10.3389/fnhum.2015.00691.

-Just for curiosity: would the outcomes be much weaker if blocks with incorrect responses were also analyzed? (I agree that there is a good reason why they should not be included.)

- If your control motor task was diametrically different, I would expect greatest sensitivity to mismatch after accounting for motor processing.

There were also grammatical comments which you may take account.

Line 383: I got lost in “as was a significant interaction of incongruency x property (ISh + ITx &gt; CSh + Ctx).

Line 58: in “(chorollary discharge)” ‘chorollary’ should read ‘corollary’, i.e., (corollary discharge).

Line 101 (and in few more places): “orally presented” sounds strange; “vocally presented” would sound a bit better. Alternatively, in a sentence such as “ ... was orally presented and an auditory cue indicated ...” ‘orally’ could just go.

In few places “SMG gyrus” should read “SMG”, as the acronym already contains the “gyrus” bit.

“spheres were constructed” should read “spheres were created”

“were also not significant” should read “were not significant, either”

## References

[B1] Allen M, Fardo F, Dietz MJ, Hillebrandt H, Friston KJ, Rees G, Roepstorff A (2016) Anterior insula coordinates hierarchical processing of tactile mismatch responses. Neuroimage 127:34–43. 10.1016/j.neuroimage.2015.11.030 26584870 PMC4758822

[B2] Amedi A, Malach R, Hendler T, Peled S, Zohary E (2001) Visuo-haptic object-related activation in the ventral visual pathway. Nat Neurosci 4:324–330. 10.1038/85201 11224551

[B3] Ashburner J, Friston KJ (2005) Unified segmentation. Neuroimage 26:839–851. 10.1016/j.neuroimage.2005.02.018 15955494

[B4] Baumgaertner A, Weiller C, Büchel C (2002) Event-related fMRI reveals cortical sites involved in contextual sentence integration. Neuroimage 16:736–745. 12169257 10.1006/nimg.2002.1134

[B5] Blakemore SJ, Smith J, Steel R, Johnstone EC, Frith CD (2000) The perception of self-produced sensory stimuli in patients with auditory hallucinations and passivity experiences: evidence for a breakdown in self-monitoring. Psychol Med 30:1131–1139. 10.1017/S0033291799002676 12027049

[B6] Bodegård A, Geyer S, Grefkes C, Zilles K, Roland PE (2001) Hierarchical processing of tactile shape in the human brain. Neuron 31:317–328. 11502261 10.1016/s0896-6273(01)00362-2

[B7] Bohlhalter S, Fretz C, Weder B (2002) Hierarchical versus parallel processing in tactile object recognition: a behavioural-neuroanatomical study of aperceptive tactile agnosia. Brain 125:2537–2548. 12390978 10.1093/brain/awf245

[B8] Botvinick MM, Braver TS, Barch DM, Carter CS, Cohen JD (2001) Conflict monitoring and cognitive control. Psychol Rev 108:624–652. 11488380 10.1037/0033-295x.108.3.624

[B9] Bursztyn LL, Ganesh G, Imamizu H, Kawato M, Flanagan JR (2006) Neural correlates of internal-model loading. Curr Biol 16:2440–2445. 10.1016/j.cub.2006.10.051 17174919

[B10] Crapse TB, Sommer MA (2008) Corollary discharge across the animal kingdom. Nat Rev Neurosci 9:587–600. 10.1038/nrn2457 18641666 PMC5153363

[B11] Desmurget M, Reilly KT, Richard N, Szathmari A, Mottolese C, Sirigu A (2009) Movement intention after parietal cortex stimulation in humans. Science 324:811–813. 10.1126/science.1169896 19423830

[B12] Eck J, Kaas AL, Goebel R (2013) Crossmodal interactions of haptic and visual texture information in early sensory cortex. Neuroimage 75:123–135. 10.1016/j.neuroimage.2013.02.075 23507388

[B13] Emmorey K, Mehta S, Grabowski TJ (2007) The neural correlates of sign versus word production. Neuroimage 36:202–208. 10.1016/j.neuroimage.2007.02.040 17407824 PMC1987366

[B14] Feinberg I (1978) Efference copy and corollary discharge: implications for thinking and its disorders. Schizophr Bull 4:636–640. 734369 10.1093/schbul/4.4.636

[B15] Flandin G, Friston KJ (2017) Analysis of family-wise error rates in statistical parametric mapping using random field theory. Hum Brain Mapp. Advance online publication. Retrieved November 1, 2017. 10.1002/hbm.23839 PMC6585687 29091338

[B16] Frankenstein U, Wennerberg A, Richter W, Bernstein C, Morden D, Rémy F, Mcintyre M (2003) Activation and deactivation in blood oxygenation level dependent functional magnetic resonance imaging. Concept Magnetic Res Educ, pp 63–70. Hoboken, New Jersey: John Wiley & Sons.

[B17] Friston KJ, Williams S, Howard R, Frackowiak RS, Turner R (1996) Movement-related effects in fMRI time-series. Magn Reson Med 35:346–355. 8699946 10.1002/mrm.1910350312

[B18] Friston KJ, Buechel C, Fink GR, Morris J, Rolls E, Dolan RJ (1997) Psychophysiological and modulatory interactions in neuroimaging. Neuroimage 6:218–229. 10.1006/nimg.1997.0291 9344826

[B19] Frith CD (2014) The cognitive neuropsychology of schizophrenia. New York, NY: Psychology Press.

[B20] Gaebler AJ, Mathiak K, Koten JW Jr, König AA, Koush Y, Weyer D, Depner C, Matentzoglu S, Edgar JC, Willmes K, Zvyagintsev M (2015) Auditory mismatch impairments are characterized by core neural dysfunctions in schizophrenia. Brain 138:1410–1423. 10.1093/brain/awv049 25743635 PMC5963408

[B21] Gitelman DR, Penny WD, Ashburner J, Friston KJ (2003) Modeling regional and psychophysiologic interactions in fMRI: the importance of hemodynamic deconvolution. Neuroimage 19:200–207. 10.1016/S1053-8119(03)00058-2 12781739

[B22] Giummarra MJ, Gibson SJ, Georgiou-Karistianis N, Bradshaw JL (2008) Mechanisms underlying embodiment, disembodiment and loss of embodiment. Neurosci Biobehav Rev 32:143–160. 10.1016/j.neubiorev.2007.07.001 17707508

[B23] Gorgolewski KJ, Varoquaux G, Rivera G, Schwartz Y, Ghosh SS, Maumet C, Sochat VV, Nichols TE, Poldrack RA, Poline J-B, Yarkoni T, Margulies DS (2015) NeuroVault.org: a web-based repository for collecting and sharing unthresholded statistical maps of the brain. Front Neuroinform 9:8. 25914639 10.3389/fninf.2015.00008 PMC4392315

[B24] Gurtubay-Antolin A, Rodriguez-Herreros B, Rodriguez-Fornells A (2015) The speed of object recognition from a haptic glance: event-related potential evidence. J Neurophysiol 113:3069–3075. 10.1152/jn.00836.2014 25744887 PMC4455565

[B25] Huntenburg JM, Bazin PL, Margulies DS (2018) Large-scale gradients in human cortical organization. Trends Cogn Sci 22:21–31. 10.1016/j.tics.2017.11.002 29203085

[B26] Johansson RS, Westling G (1987) Signals in tactile afferents from the fingers eliciting adaptive motor responses during precision grip. Exp Brain Res 66:141–154. 3582528 10.1007/BF00236210

[B57] Jenmalm P, Schmitz C, Forssberg H, Ehrsson HH (2006) Lighter or heavier than predicted: neural correlates of corrective mechanisms during erroneously programmed lifts. Journal of Neuroscience 26(35):9015–9021. 10.1523/jneurosci.5045-05.2006 16943559 PMC6675347

[B58] Jessup RK, Busemeyer JR, Brown JW (2010) Error effects in anterior cingulate cortex reverse when error likelihood is high. J Neurosci 30:3467–3472. 20203206 10.1523/JNEUROSCI.4130-09.2010 PMC2841347

[B27] Johansson RS, Westling G (1988) Coordinated isometric muscle commands adequately and erroneously programmed for the weight during lifting task with precision grip. Exp Brain Res 71:59. 71:3416958 10.1007/BF00247522

[B28] Johansson RS, Cole KJ (1992) Sensory-motor coordination during grasping and manipulative actions. Curr Opin Neurobiol 2:815–823. 1477545 10.1016/0959-4388(92)90139-c

[B29] Johansson RS, Birznieks I (2004) First spikes in ensembles of human tactile afferents code complex spatial fingertip events. Nat Neurosci 7:170–177. 10.1038/nn1177 14730306

[B30] Johansson RS, Flanagan JR (2008) Tactile sensory control of object manipulation in humans. The senses: a comprehensive reference, pp 67–86. New York, NY: Academic Press.

[B31] Kaas AL, Van Mier H, Goebel R (2007) The neural correlates of human working memory for haptically explored object orientations. Cereb Cortex 17:1637–1649. 10.1093/cercor/bhl074 16966490

[B32] Kassuba T, Menz MM, Röder B, Siebner HR (2013) Multisensory interactions between auditory and haptic object recognition. Cereb Cortex 23:1097–1107. 10.1093/cercor/bhs076 22518017

[B33] Kriegeskorte N, Simmons WK, Bellgowan PS, Baker CI (2008) Circular inference in neuroscience: the dangers of double dipping. J Vis 8:88 10.1167/8.6.88 PMC2841687 19396166

[B34] Lau EF, Phillips C, Poeppel D (2008) A cortical network for semantics:(de) constructing the N400. Nat Rev Neurosci 9:920–933. 10.1038/nrn2532 19020511

[B35] Malekshahi R, Seth A, Papanikolaou A, Mathews Z, Birbaumer N, Verschure PF, Caria A (2016) Differential neural mechanisms for early and late prediction error detection. Sci Rep 6:24350. 27079423 10.1038/srep24350 PMC4832139

[B59] Monaco S, Gallivan JP, Figley TD, Singhal A, Culham JC (2017) Recruitment of foveal retinotopic cortex during haptic exploration of shapes and actions in the dark. JNeurosci 37:11572–11591. 29066555 10.1523/JNEUROSCI.2428-16.2017 PMC6705744

[B36] Marangon M, Kubiak A, Króliczak G (2016) Haptically guided grasping. fMRI shows right-hemisphere parietal stimulus encoding, and bilateral dorso-ventral parietal gradients of object- and action-related processing during grasp execution. Front Hum Neurosci 9:691. 10.3389/fnhum.2015.00691 26779002 PMC4700263

[B37] Naito E, Roland PE, Grefkes C, Choi HJ, Eickhoff S, Geyer S, Zilles K, Ehrsson HH (2005) Dominance of the right hemisphere and role of area 2 in human kinesthesia. J Neurophysiol 93:1020–1034. 10.1152/jn.00637.2004 15385595

[B38] Nee DE, Kastner S, Brown JW (2011) Functional heterogeneity of conflict, error, task-switching, and unexpectedness effects within medial prefrontal cortex. Neuroimage 54:528–540. 10.1016/j.neuroimage.2010.08.027 20728547 PMC2962721

[B60] Oliveira FT, McDonald JJ, Goodman D (2007) Performance monitoring in the anterior cingulate is not all error related: expectancy deviation and the representation of action-outcome associations. J Cogn Neurosci 19:1994–2004. 17892382 10.1162/jocn.2007.19.12.1994

[B39] Padrao G, Gonzalez-Franco M, Sanchez-Vives MV, Slater M, Rodriguez-Fornells A (2016) Violating body movement semantics: neural signatures of self-generated and external-generated errors. Neuroimage 124:147–156. 10.1016/j.neuroimage.2015.08.022 26282856

[B40] Perrett DI, Harris MH, Mistlin AJ, Hietanen JK, Benson PJ, Bevan R, Thomas S, Oram MW, Ortega J, Brierly K (1990) Social signals analyzed at the single cell level: someone is looking at me something moved! Int J Comp Psychol 4:25–55.

[B41] Ripollés P, Marco-Pallarés J, Hielscher U, Mestres-Missé A, Tempelmann C, Heinze HJ, Rodríguez-Fornells A, Noesselt T (2014) The role of reward in word learning and its implications for language acquisition. Curr Biol 24:2606–2611. 10.1016/j.cub.2014.09.044 25447993

[B42] Ripollés P, Marco-Pallares J, Alicart H, Tempelmann C, Rodriguez-Fornells A, Noesselt T (2016) Intrinsic monitoring of learning success facilitates memory encoding via the activation of the SN/VTA-Hippocampal loop. Elife 5. 10.7554/eLife.17441 PMC5030080 27644419

[B43] Roland PE, O'Sullivan B, Kawashima R (1998) Shape and roughness activate different somatosensory areas in the human brain. Proc Natl Acad Sci USA 95:3295–3300. 10.1073/pnas.95.6.3295 9501256 PMC19735

[B44] Romo R, Brody CD, Hernández A, Lemus L (1999) Neuronal correlates of parametric working memory in the prefrontal cortex. Nature 399:470–473. 10.1038/20939 10365959

[B45] Rumiati RI, Weiss PH, Shallice T, Ottoboni G, Noth J, Zilles K, Fink GR (2004) Neural basis of pantomiming the use of visually presented objects. Neuroimage 21:1224–1231. 10.1016/j.neuroimage.2003.11.017 15050550

[B46] Rushworth MF, Walton ME, Kennerley SW, Bannerman DM (2004) Action sets and decisions in the medial frontal cortex. Trends Cogn Sci 8:410–417. 10.1016/j.tics.2004.07.009 15350242

[B47] Sathian K (2016) Analysis of haptic information in the cerebral cortex. J Neurophysiol 116:1795–1806. 10.1152/jn.00546.2015 27440247 PMC5144710

[B48] Sathian K, Lacey S, Stilla R, Gibson GO, Deshpande G, Hu X, Laconte S, Glielmi C (2011) Dual pathways for haptic and visual perception of spatial and texture information. Neuroimage 57:462–475. 10.1016/j.neuroimage.2011.05.001 21575727 PMC3128427

[B49] Sirigu A, Daprati E, Ciancia S, Giraux P, Nighoghossian N, Posada A, Haggard P (2004) Altered awareness of voluntary action after damage to the parietal cortex. Nat Neurosci 7:80–84. 10.1038/nn1160 14647290

[B50] Von Holst E, Mittelstaedt H (1950) The principle of reafference. Naturwissenschaften 37:464–476. 10.1007/BF00622503

[B51] Wang L, Li X, Hsiao SS, Bodner M, Lenz F, Zhou YD (2012) Behavioral choice-related neuronal activity in monkey primary somatosensory cortex in a haptic delay task. J Cogn Neurosci 24:1634–1644. 10.1162/jocn_a_00235 22452554 PMC4034389

[B52] Wolpert DM, Miall RC (1996) Forward models for physiological motor control. Neural Netw 9:1265–1279. 12662535 10.1016/s0893-6080(96)00035-4

[B53] Wolpert DM, Miall RC, Kawato M (1998) Internal models in the cerebellum. Trends Cogn Sci 2:338–347. 21227230 10.1016/s1364-6613(98)01221-2

[B54] Woo CW, Krishnan A, Wager TD (2014) Cluster-extent based thresholding in fMRI analyses: pitfalls and recommendations. Neuroimage 91:412–419. 10.1016/j.neuroimage.2013.12.058 24412399 PMC4214144

[B55] Xu X, Yuan H, Lei X (2016) Activation and connectivity within the default mode network contribute independently to future-oriented thought. Sci Rep 6:21001. 10.1038/srep21001 26867499 PMC4751480

[B56] Yarkoni T, Poldrack RA, Nichols TE, Van Essen DC, Wager TD (2011) Large-scale automated synthesis of human functional neuroimaging data. Nat Methods 8:665–670. 10.1038/nmeth.1635 21706013 PMC3146590

